# Inter-rater agreement of the Quality of Life-Alzheimer’s Disease (QoL-AD) self-rating and proxy rating scale: secondary analysis of RightTimePlaceCare data

**DOI:** 10.1186/s12955-018-0959-y

**Published:** 2018-06-28

**Authors:** Josephine Römhild, Steffen Fleischer, Gabriele Meyer, Astrid Stephan, Sandra Zwakhalen, Helena Leino-Kilpi, Adelaida Zabalegui, Kai Saks, Maria Soto-Martin, Caroline Sutcliffe, Ingalill Rahm Hallberg, Almuth Berg, on behalf of the RightTimePlaceCare Consortium

**Affiliations:** 1 Institute of Health and Nursing Science, Medical Faculty, Martin Luther University Halle-Wittenberg, Halle, Germany; 2 Institute of General Practice and Family Medicine, Jena University Hospital, Jena, Germany; 3 School of Nursing Science, Witten/Herdecke University, Witten, Germany; 4 Department of Health Services Research, Care and Public Health Research Institute (CAPHRI), Maastricht University, Maastricht, Netherlands; 5 Department of Nursing Science, Faculty of Medicine, University of Turku and Turku University Hospital, Turku, Finland; 6 Hospital Clinic of Barcelona, Barcelona, Spain; 7 Department of Internal Medicine, Faculty of Medicine, University of Tartu, Tartu, Estonia; 8 Geriatrics Department, Gerontôpole, Toulouse University Hospital, INSERM UMR 1027, Toulouse, France; 9 School of Health Sciences, Faculty of Biology, Medicine and Health, University of Manchester, Manchester, UK; 10 Department of Health Sciences, Medical Faculty, Lund University, Lund, Sweden

**Keywords:** Dementia, Quality of life, Psychometrics, Reliability, Inter-rater agreement

## Abstract

**Background:**

To assess the quality of life of people with dementia, measures are required for self-rating by the person with dementia, and for proxy rating by others. The Quality of Life in Alzheimer’s Disease scale (QoL-AD) is available in two versions, QoL-AD-SR (self-rating) and QoL-AD-PR (proxy rating).

The aim of our study was to analyse the inter-rater agreement between self- and proxy ratings, in terms of both the total score and the items, including an analysis specific to care setting, and to identify factors associated with this agreement.

**Methods:**

Cross-sectional QoL-AD data from the 7th Framework European RightTimePlaceCare study were analysed. A total of 1330 cases were included: *n* = 854 receiving home care and *n* = 476 receiving institutional long-term nursing care. The proxy raters were informal carers (home care) and best-informed professional carers (institutional long-term nursing care).

Inter-rater agreement was investigated using Bland-Altman plots for the QoL-AD total score and by weighted kappa statistics for single items. Associations were investigated by regression analysis.

**Results:**

The overall QoL-AD assessment of those with dementia revealed a mean value of 33.2 points, and the proxy ratings revealed a mean value of 29.8 points.

The Bland-Altman plots revealed a poor agreement between self- and proxy ratings for the overall sample and for both care settings. With one exception (item ‘Marriage’ weighted kappa 0.26), the weighted kappa values for the single QoL-AD items were below 0.20, indicating poor agreement.

Home care setting, dementia-related behavioural and psychological symptoms, and the functional status of the person with dementia, along with the caregiver burden, were associated with the level of agreement. Only the home care setting was associated with an increase larger than the predefined acceptable difference between self- and proxy ratings.

**Conclusions:**

Proxy quality of life ratings from professional and informal carers appear to be lower than the self-ratings of those with dementia.

QoL-AD-SR and QoL-AD-PR are therefore not interchangeable, as the inter-rater agreement differs distinctly. Thus, a proxy rating should be judged as a complementary perspective for a self-assessment of quality of life by those with dementia, rather than as a valid substitute.

**Electronic supplementary material:**

The online version of this article (10.1186/s12955-018-0959-y) contains supplementary material, which is available to authorized users.

## Background

Improving quality of life (QoL) is an important focus of various therapeutic interventions for people with dementia [1]. Therefore, QoL is increasingly gaining importance as an outcome measure to evaluate interventions in dementia care [2]. The QoL of those with dementia is considered as an individual, subjective, dynamic, multidimensional and complex construct, which includes the assessment of and adaptation to the consequences of dementia [3–6]. QoL can only be understood within the person-environment system, which Lawton has described in four sectors: behavioural competence (evaluated functioning of a person in relation to inner or outer events), perceived QoL (evaluations about major life domains), psychological well-being, and objective environment [6, 7].

The measurement of dementia-specific QoL involves particular features, due to the particular symptoms of the disease. Functional impairment of memory, cognition, time perception, attention, judgement and communication, along with the level of insight into the illness and the severity of the disease can all influence how questions are understood, and the rating and communication of the subjective condition [8–12]. In addition, the reliability of QoL assessment is affected by limitations of consciousness [13] and behavioural and non-cognitive symptoms such as depression, restlessness and psychosis [8]. Emotional symptoms such as social deprivation can also influence QoL ratings by people with dementia [9]. The progress of dementia is likely to make self-rating (SR) infeasible at a certain point. For people with severe dementia, proxy rating (PR) by informal or professional carers is indispensable if they are not to be excluded from QoL determination.

A recent review [13] has identified 16 QoL measures for people with dementia and has assessed their psychometric properties as well. Many measures were based on proxy assessment, with questionable validity for people with mild to moderate dementia. The best researched measure was the Quality of Life in Alzheimer’s Disease scale (QoL-AD) [14]. It is available as a self-rating version (QoL-AD-SR: Quality of Life in Alzheimer’s Disease Self-Rating scale) and as a proxy rating version (QoL-AD-PR: Quality of Life in Alzheimer’s Disease Proxy Rating scale). The instrument has been translated in various languages and has good psychometric properties overall [14]. Bowling et al. [13] give an overview of the psychometric evidence. However, some discrepancy between the two rating versions has been identified, which indicates further research aimed to clarify the relationship between the assessment through self-rating by the person with dementia and proxy rating [13].

We conducted a systematic literature search in Medline via PubMed (April 2016), which was aimed at identifying the body of knowledge on the agreement between the two QoL-AD rating versions. The search strategy used was: ‘*QoL-AD OR (quality of life Alzheimer’s disease) OR (quality of life Alzheimer’s disease scale) OR (quality of life Alzheimer’s disease questionnaire) AND ((agreement OR accordance OR consensus OR conformity OR rapport OR congruence OR match) OR (discrepancy OR gap OR mismatch OR difference OR distinction OR disagreement) OR (caregiver bias)) AND (self OR (proxy OR caregiver OR carer))’.* According to the four-phase PRISMA [15] process for selecting articles, all studies in the English or German language published in the last 10 years were included. A total of 28 relevant studies remained [16–43]. Across these studies, carers assessed QoL lower than those suffering from dementia. Fourteen articles [19, 20, 22, 25, 26, 28, 29, 31, 33, 36, 37, 39–41] reported the level of agreement between QoL-AD self- and proxy ratings; ranging from a poor [20, 28] to a very good [37] agreement. In almost all of the studies, the level of agreement at the QoL-AD total score was estimated using correlation analyses, such as the calculation of the intraclass correlation coefficient (ICC) or the application of the paired-samples t-test. However, calculation of correlation coefficients is never appropriate for agreement assessment, as correlation coefficients give information about a linear relation of two metric variables and not about the agreement [44, 45]. Two clearly distinct measures that are intended to assess the same construct regularly correlate in a certain way without necessarily reaching good agreement and good agreement can be reached without good correlation. Interpreting correlation analyses as agreement assessment must therefore be questioned and should generally be avoided. Only two studies [25, 28] used the Bland-Altman plots as recommended for metric variables [44, 45]; the presented data indicate an unacceptable range of agreement. Factors associated with the level of agreement between self- and proxy ratings were reported in eleven studies [16–22, 25, 27, 28, 36]. The most frequently mentioned factors for different ratings were behavioural and psychological symptoms of dementia (BPSD) and severity of cognitive impairment of those with dementia, as well as caregiver burden (see Additional file 1).

We found no studies investigating the level of agreement between QoL-AD-SR and QoL-AD-PR or agreement at the item level in a large European sample.

Therefore, our study aims to explore the inter-rater agreement of the measures QoL-AD-SR and QoL-AD-PR with the following objectives:

1. To investigate inter-rater agreement at the total score level.
2. To investigate inter-rater agreement on the item level.
3. To investigate inter-rater agreement by comparison of care settings (institutional long-term nursing care versus formal home care).

In addition, the identified factors (see Additional file 1) were hypothesised to be associated with the level of agreement and explored using regression analysis.

## Methods

Data for the secondary analysis were obtained from the European RightTimePlaceCare study (RTPC; FP7-Health-F3–2010-242,153) [46]. Cross-sectional data were used.

### The RTPC study

The RTPC study comprised a prospective, multi-centre cohort study in eight European countries: Estonia, Finland, France, Germany, the Netherlands, Spain, Sweden and the UK. The survey (start in 2010) generated primary data for the development of best-practice recommendations on the transition from home care to institutional nursing care for European citizens with dementia.

Two types of dyads of people with dementia and their informal carers were investigated [46]:

1. People with dementia (and their informal carers) living in an institutional long-term nursing care (ILTC) facility, admitted one to 3 months ago;
2. People with dementia (and their informal carers) living at home receiving professional long-term home nursing care (HC), who were assessed as being at risk of institutionalisation within the next 6 months.

The RTPC inclusion criteria for those with dementia were (1) a formal diagnosis of dementia; (2) aged ≥65 years; (3) a Standardized Mini Mental-State Examination (S-MMSE) [47] score of ≤24; (4) no primary psychiatric diagnosis or Korsakoff syndrome; and (5) personal contact with their informal carer at least twice a month. Those most involved in caring for the person with dementia were included as informal carers. No restrictions on their relationships with the person with dementia were imposed, but those who provide care as volunteers were excluded [46]. Detailed information about the RTPC study design has been published elsewhere [46].

The RTPC study sample consisted of 2014 people with dementia (and their informal carers) [42]: 791 dyads in the ILTC sample (from 256 ILTC locations) and 1223 dyads in the HC sample.

### Variables from the RTPC data set for the secondary analysis

#### QoL-AD measure

To analyse the inter-rater agreement, baseline data of the QoL-AD self- and proxy ratings are important. Therefore, only cases with total scores available for both the QoL-AD-SR and the QoL-AD-PR were included in our secondary data analysis. In total, *n* = 1330 cases were selected (see Additional file 2).

The QoL of people with dementia was assessed by self- and proxy ratings in all eight countries [46] using the 13-item version of the QoL-AD [14]. The self-rating was assessed by those with dementia if they had an S-MMSE score of three or higher. The QoL-AD proxy rating is assessed from the proxy’s own perspective (proxy-proxy perspective), in contrast to answering as the patient would (person-proxy perspective). The proxy report was obtained from the best informed proxy, i.e. QoL in HC was assessed by informal carers and by professional carers in ILTC (i.e., nursing staff).

Data were collected between November 2010 and April 2012. To standardise the data collection, an instruction manual [48] was provided and implemented through training. This included instructions for the face-to-face interviews of the QoL-AD according to the detailed instructions for interviewers in the original questionnaire. The countries’ main investigators were responsible for the training of the interviewers; all interviewers received training regarding the content and completion of questionnaires [46].

The total scores of the self- and proxy ratings were calculated according to the specifications of the original authors [8, 14]: Based on the four response categories (1 = poor, 2 = fair, 3 = good, 4 = excellent) of the 13 items, the total score ranges from 13 to 52, with higher values indicating a higher QoL. If two or fewer responses were missing, they were replaced by the mean of the remaining individual responses. If more than two responses were missing, no total score was calculated.

#### Variables for the analysis of associated factors on the agreement

To analyse associated factors on the agreement of the self- and proxy ratings relevant clinical variables were selected from the RTPC data set. Our selection criteria were guided by the findings from the systematic literature search concerning the factors associated with the level of agreement between self- and proxy ratings. The corresponding variables from the RTPC data set are presented in detail in Additional file 1, including description, value ranges, and interpretation of the measures.

Variables of people with dementia were BPSD measured by the *Neuropsychiatric Inventory Questionnaire (NPI-Q)* [49, 50], cognitive function/severity of dementia measured by the *Standardized Mini Mental-State Examination (S-MMSE)* [47, 51] (sometimes used as a surrogate method for staging dementia [52]), depression measured by the *Cornell Scale for Depression in Dementia (CSDD)* [53], functional status measured by the *Katz Index of Independence in Activities of Daily Living (KATZ ADL)* [54], educational background, and care setting.

Variables of informal carers were caregiver burden measured by the *Zarit Burden Inventory (ZBI)* [55], the subscale ‘Lack of family support’ of the *Caregiver Reaction Assessment (CRA)* [56], and the *Neuropsychiatric Inventory Questionnaire-Caregiver Distress (NPI-Q-D)* [49, 50], QoL measured by the *European Quality of Life Scale (EQ-5D-3 L, EQ-5D VAS)* [57, 58], the *General Health Questionnaire 12-item version (GHQ-12)* [59], and the subscale ‘Impact on health’ of the *Caregiver Reaction Assessment (CRA)* [56], kind of relationship to the person with dementia, and gender.

Sociodemographic or clinical variables of the professional carers relevant to the secondary data analysis were not collected in the RTPC study.

### Statistical analysis

Descriptive data from people with dementia, informal carers and professional carers were analysed using the statistical software SPSS Version 24. Frequencies, proportions, means and standard deviations were calculated.

With reference to the descriptive methods recommended for metric variables [44, 60], we took an exploratory approach for the assessment of the agreement between self- and proxy ratings of the QoL-AD total score and used Bland-Altman plots [44, 61] created with the statistical software R Version 3.2.

Bland-Altman plots usually consist of a line representing the mean of all differences of the compared methods and the Limits of Agreement (LoA). We initially decided that an acceptable difference between the self- and proxy rating of the QoL-AD would be within a range of − 3 to + 3 points in the total score. These boundaries correspond to a difference of half a standard deviation [43], which is generally judged to be of minimum clinical importance for QoL measurements [62]. For a more comprehensive impression of Bland-Altman plots we added lines for the standard deviation of the differences, and the boundaries of an acceptable difference (+/− 3 scale points).

To investigate inter-rater agreement on the item level Cohen’s weighted kappa statistic [63, 64] and corresponding 95% confidence intervals (95% CI) were calculated using the statistical software R Version 3.2. Before calculation, imputed values were removed from the data. The interpretation of kappas was guided by Altman’s recommendation [65]: ≤ 0.20 poor, 0.21–0.40 fair, 0.41–0.60 moderate, 0.61–0.80 good, 0.81–1.0 very good.

The analysis of covariance was used to analyse associated factors on the agreement of the self- and proxy ratings. In the subpopulation of people with dementia living in ILTC, the variables of informal carers were also assessed, but they did not rate the QoL-AD for those with dementia as proxies. Thus, we assumed that they would have no influence on the level of agreement. Therefore, two predefined models were fitted, including the aforementioned variables (see Subsection ‘Variables for the analysis of associated factors on the agreement’) as independent variables and the difference between self- and proxy ratings as the dependent variable (self-rating minus proxy rating). Model 1 included all people with dementia (ILTC and HC settings), with no further variables on the informal carers (as they did not do the proxy rating in ILTC). Model 2 included people with dementia in the HC setting, with variables on the informal carers (proxy raters). Both models were fitted in two ways: a) using the original values of the scales – the resulting coefficients can be interpreted in comparison with the used scales (partial regression coefficient: B); b) transforming all scales to a standardised version (z-transformation) – the resulting standardised partial regression coefficients can be interpreted in a similar way to a standardised effect size, which makes different scales more easily comparable (standardised partial regression coefficient: β). The analysis of covariance was performed using the statistical software R Version 3.2.

### Ethical and legal aspects

Ethical approval was obtained from country-specific legal authorities for research on human beings. Written informed consent was obtained from all participants, from the (legal) representatives and whenever possible from the people with dementia themselves [46].

The RTPC consortium approved and released the data set.

## Results

### Study sample description

A total of *n* = 1330 persons with dementia assessed their own QoL (see Table 1). The majority were female (65.9%) and lived at home (64.2%). Their mean age was 83.0 years. In the home care setting, the majority of those with dementia were married (49.3%), while the majority with dementia living in ILTC were widowed (61.7%).

**Table 1:** Characteristics of people with dementia

	Total *n* = 1330	ILTC *n* = 476 (35.8%)	HC *n* = 854 (64.2%)
Age/ years, mean (SD)	83.0 (6.4)	84.4 (6.1)	82.3 (6.4)
Gender, n (%)			
Female	876 (65.9%)	346 (72.7%)	530 (62.1%)
Male	454 (34.1%)	130 (27.3%)	324 (37.9%)
Marital status, n (%)			
Married	549 (41.3%)	128 (26.9%)	421 (49.3%)
Widowed	662 (49.8%)	293 (61.7%)	369 (43.2%)
Divorced	69 (5.2%)	31 (6.5%)	38 (4.4%)
Other	49 (3.7%)	23 (4.8%)	26 (3.0%)
Length of formal education/ years, mean (SD)	9.1 (3.8)	8.9 (3.6)	9.1 (3.9)
NPI-Q, mean (SD)	7.9 (6.0)	6.3 (5.5)	8.8 (6.1)
S-MMSE, mean (SD)	15.0 (5.7)	13.3 (5.5)	15.9 (5.6)
CSDD, mean (SD)	6.9 (5.5)	5.5 (4.7)	7.7 (5.7)
KATZ ADL, mean (SD)	3.4 (1.8)	2.6 (1.7)	3.8 (1.7)

For some variables, data are not available for all participants (percentages are relative frequencies of the valid values)

Value ranges of measures: see Additional file 1

For those with dementia living in ILTC, *n* = 476 professional carers assessed their QoL (see Table 2). The average age of the carers was 41.9 years. One third was registered nurses (33.3%) and more than half were trained nursing assistants (55.9%). The proxy ratings for people with dementia living at home were assessed by *n* = 854 informal carers (see Table 2) with an average age of 64.8 years. The informal carers were predominantly female (69.2%), adult children (45.1%) and were married (77.3%).

**Table 2:** Characteristics of proxy raters

ILTC: Professional carers (n = 476)	
Age/ years, mean (SD)	41.9 (11.8)
Weekly hours of work, mean (SD)	35.7 (5.6)
Professional education, n (%)	
Nursing aid	48 (10.7%)
Certified nursing assistant	250 (55.9%)
Registered nurse	149 (33.3%)
HC: Informal carers (n = 854)	
Age/ years, mean (SD)	64.8 (13.5)
Gender, n (%)	
Female	590 (69.2%)
Male	262 (30.8%)
Marital status, n (%)	
Married/cohabitant	660 (77.3%)
Widowed	42 (4.9%)
Divorced	85 (10.0%)
Never married	67 (7.8%)
Relationship to person with dementia, n (%)	
Husband	142 (16.6%)
Wife	208 (24.4%)
Child	385 (45.1%)
Friend	12 (1.4%)
Other	106 (12.4%)
ZBI, mean (SD)	31.7 (15.6)
CRA/ Lack of family support (subscale), mean (SD)	12.1 (4.7)
CRA/ Impact on health (subscale), mean (SD)	9.9 (3.9)
EQ-5D-3 L, mean (SD)	0.8 (0.3)
EQ-5D VAS, mean (SD)	68.7 (18.6)
GHQ-12, mean (SD)	12.8 (5.7)
NPI-Q-D, mean (SD)	10.6 (9.0)

For some variables, data are not available for all participants (percentages are relative frequencies of the valid values)

Value ranges of measures: see Additional file 1

The overall QoL-AD assessment of those with dementia revealed a mean value of 33.2 out of 52 points (see Table 3). The average self-rating in the ILTC setting was 32.5 points and in the HC setting it was 33.5 points. The ratings at the item level were consistently within the medium range of response categories (2.1 to 3.1 points). The items ‘Memory’ (2.1) and ‘Ability to do chores around the house’ (2.2) showed the most negative self-ratings, while the item ‘Marriage’ (3.1) showed the most positive rating. There were only minor differences between self-ratings in ILTC and HC settings (≤ 0.3 points).

**Table 3:** Self-rating and proxy rating of quality of life; QoL-AD baseline data from the RTPC study

	QoL-AD-SR, mean (SD)			QoL-AD-PR, mean (SD)		
	Total	ILTC	HC	Total	ILTC (professional carers)	HC (informal carers)
	*n* = 1330	*n* = 476	*n* = 854	*n* = 1330	*n* = 476	*n* = 854
Total score	33.2 (6.1)	32.5 (6.3)	33.5 (5.9)	29.8 (5.5)	31.5 (5.8)	28.8 (5.1)
Physical health	2.4 (0.8)	2.4 (0.8)	2.4 (0.8)	2.2 (0.8)	2.3 (0.7)	2.1 (0.8)
Energy	2.4 (0.8)	2.4 (0.8)	2.4 (0.8)	2.1 (0.8)	2.4 (0.8)	1.9 (0.8)
Mood	2.5 (0.8)	2.4 (0.8)	2.5 (0.8)	2.3 (0.7)	2.4 (0.8)	2.2 (0.7)
Living situation	2.9 (0.7)	2.7 (0.7)	3.0 (0.7)	2.9 (0.7)	2.9 (0.7)	3.0 (0.7)
Memory	2.1 (0.8)	2.1 (0.8)	2.0 (0.7)	1.5 (0.6)	1.7 (0.7)	1.3 (0.5)
Family	3.0 (0.7)	2.9 (0.8)	3.1 (0.6)	2.9 (0.8)	3.1 (0.8)	2.8 (0.8)
Marriage	3.1 (0.8)	3.0 (0.8)	3.1 (0.7)	2.9 (0.8)	3.0 (0.9)	2.9 (0.8)
Friends	2.6 (0.8)	2.6 (0.8)	2.6 (0.8)	2.3 (0.9)	2.4 (0.9)	2.2 (0.9)
Self as a whole	2.6 (0.7)	2.5 (0.8)	2.6 (0.7)	2.4 (0.8)	2.6 (0.7)	2.3 (0.8)
Ability to do chores around the house	2.2 (0.9)	2.2 (0.9)	2.2 (0.8)	1.6 (0.8)	1.7 (0.8)	1.5 (0.7)
Ability to do things for fun	2.4 (0.8)	2.3 (0.9)	2.4 (0.8)	1.9 (0.8)	2.2 (0.9)	1.7 (0.8)
Money	2.5 (0.7)	2.4 (0.8)	2.6 (0.7)	2.5 (0.8)	2.4 (0.8)	2.6 (0.8)
Life as a whole	2.6 (0.7)	2.6 (0.8)	2.7 (0.7)	2.4 (0.7)	2.5 (0.7)	2.3 (0.7)

Points are assigned to each item as follows: poor = 1, fair = 2, good = 3, excellent = 4

The overall proxy rating average was 29.8 points (see Table 3), the average in ILTC by the professional carers was 31.5 points and in the HC by the informal carers it was 28.8 points. The proxy rating on item level ranged from 1.5 to 2.9 points. The items ‘Living situation’ (2.9), ‘Family’ (2.9) and ‘Marriage’ (2.9) achieved the highest ratings, while the item ‘Memory’ (1.5) had the lowest score.

### Inter-rater agreement at the total score

**Fig. 1:**
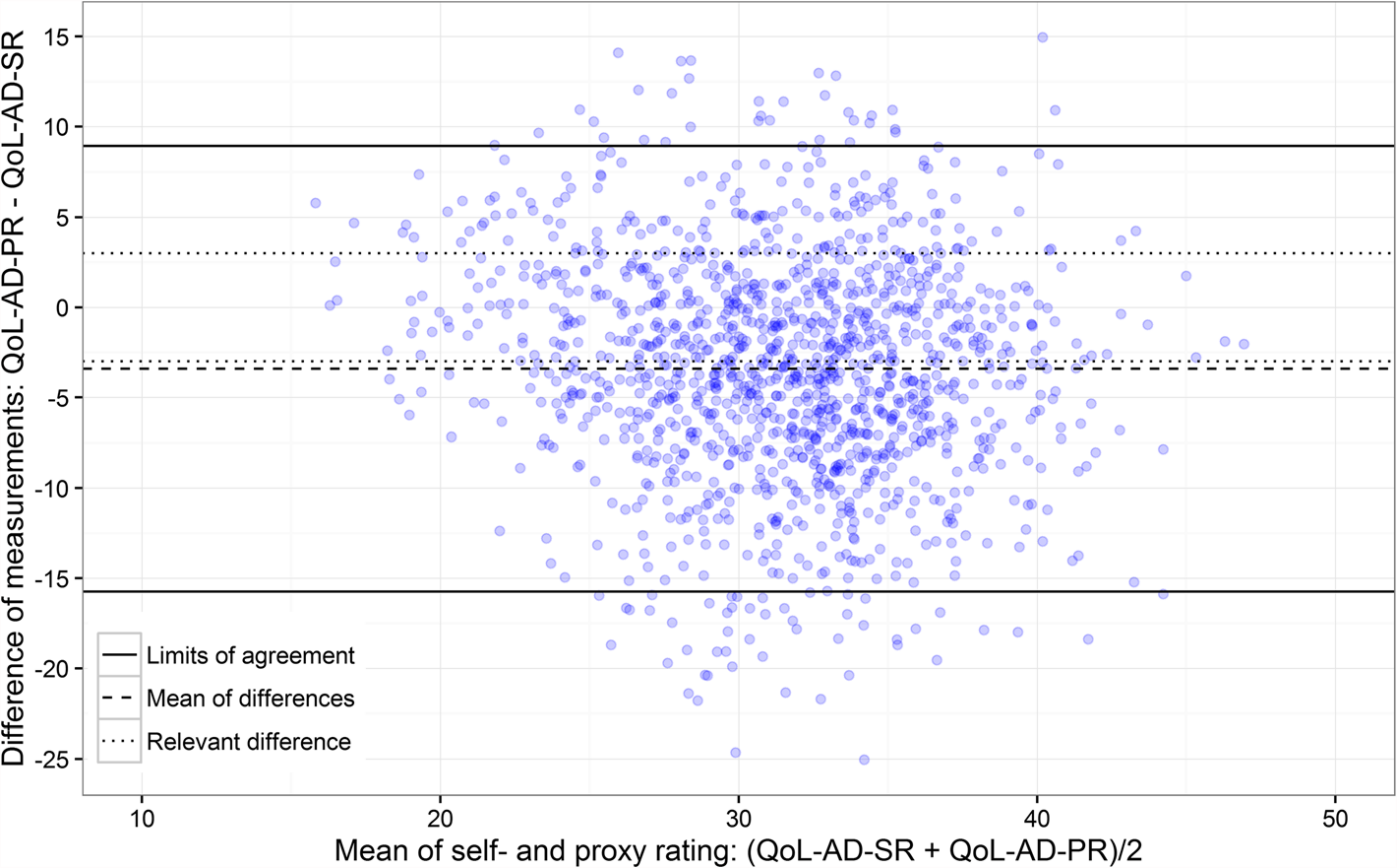
Bland-Altman plot of the inter-rater agreement for the total score; overall sample

The Bland-Altman plot (see Fig. 1) showed a mean difference of the paired observations of − 3.4, i.e., the carers’ QoL ratings of those with dementia were lower than the self-ratings of those with dementia. The upper limit of agreement (LoA) shows a difference of 8.5 scale points and the lower LoA a difference of − 15.7 scale points. Both LoA by far exceed the acceptable deviation of +/− 3 scale points previously mentioned. No clear pattern in the differences of the paired observations can be identified in the plot.

#### Inter-rater agreement by comparison of the care settings

No relevant difference was found between the self-rating and the proxy rating when the mean of the paired observations’ differences in ILTC was compared, the professional carers’ rating QoL being one point less than that of those with dementia (see Table 3). In HC a relevant difference of 4.7 mean points between the paired measurements was found, indicating that informal carers rated QoL substantially lower than those with dementia.

**Fig. 2:**
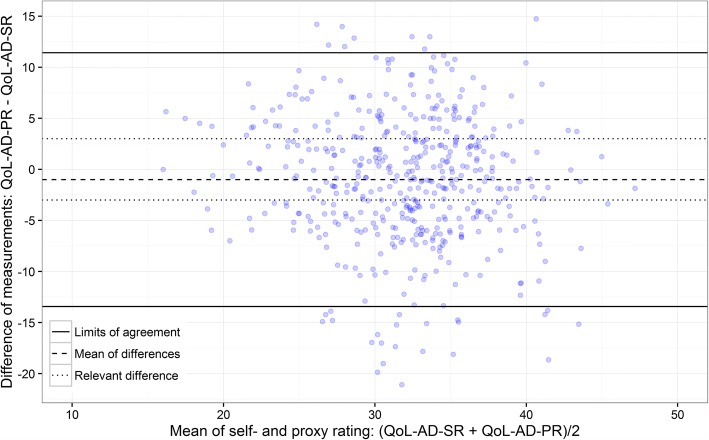
Bland-Altman plot of the inter-rater agreement for the total score; institutional long-term nursing care (ILTC) setting

**Fig. 3:**
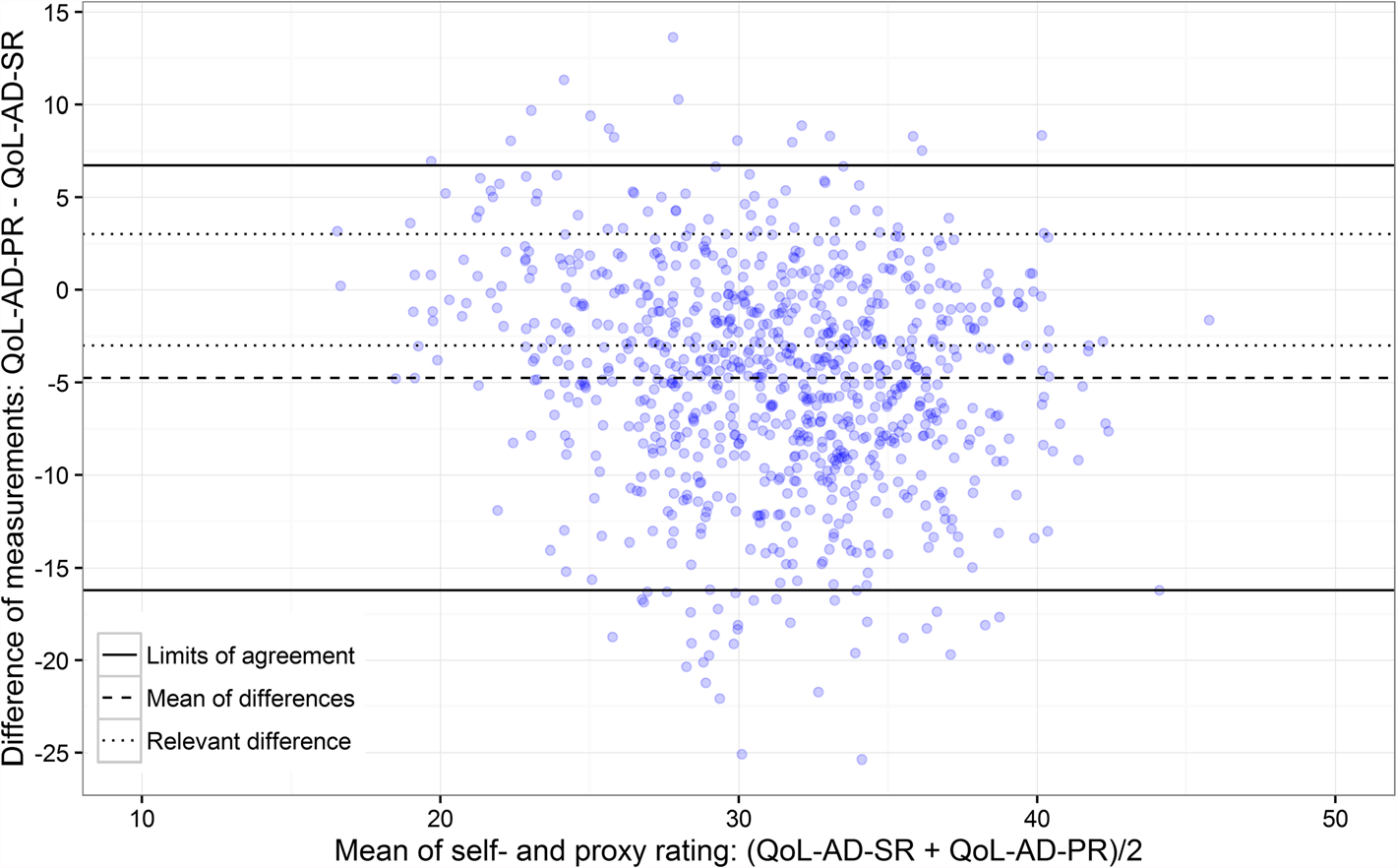
Bland-Altman plot of the inter-rater agreement for the total score; home care (HC) setting

The LoA in ILTC (see Fig. 2) ranged between − 13.4 and 11.4 scale points, far outside the acceptable deviation of +/− 3. No pattern of differences of the paired observations could be identified in this plot and, similarly, the LoA in HC (see Fig. 3) ranged between − 16.2 and 6.7 scale points, again with no pattern of differences of the paired observations.

### Inter-rater agreement on item level

The agreement of single QoL-AD items (see Table 4) revealed weighted kappas smaller than 0.20, representing poor agreement, which is in accordance with Altman [65]. Only the item ‘Marriage’ revealed fair agreement (weighted kappa: 0.26).

**Table 4:** Inter-rater agreement at the item level; weighted kappa (κ_w_) and 95% confidence intervals (95% CI)

QoL-AD item	Number of cases (n)	κ_w_	95% CI
Marriage	1232	0.26	(0.20; 0.31)
Family	1319	0.17	(0.11; 0.23)
Living situation	1315	0.15	(0.10; 0.21)
Ability to do chores around the house	1314	0.15	(0.11; 0.20)
Physical health	1328	0.11	(0.05; 0.17)
Energy	1318	0.11	(0.06; 0.17)
Mood	1317	0.11	(0.05; 0.17)
Money	1251	0.10	(0.04; 0.16)
Friends	1274	0.09	(0.03; 0.14)
Memory	1318	0.06	(0.01; 0.10)
Ability to do things for fun	1309	0.04	(−0.01; 0.10)
Self as a whole	1285	0.04	(−0.02; 0.10)
Life as a whole	1310	0.02	(−0.04; 0.08)

### Regression models of factors associated with the level of agreement

The investigated predictor variables in both regression models (see Table 5) accounted for 13 and 8% of the variance of differences between self- and proxy-assessment respectively, with the QoL-AD (Model 1: *r*^2^_adjusted_ = 0.13; Model 2: *r*^2^_adjusted_ = 0.08).

**Table 5:** Associated factors on the agreement of self- and proxy ratings; partial regression coefficients (B) and standardised partial regression coefficients (β)

		Model 1 (total sample: ILTC + HC) *r*^2^_adjusted_ = 0.13					Model 2 (subsample: HC setting) *r*^*2*^_adjusted_ = 0.08			
Factor	Measure/ variable	B		95% CI	ß	95% CI	B	95% CI	ß	95% CI
Intercept		1.09			−0.35		2.75		0.23	
People with dementia										
Behavioural and psychological symptoms of dementia	NPI-Q		**0.21**	**(0.13; 0.29)**	**0.20**	**(0.12; 0.28)**	**0.30**	**(0.15; 0.44)**	**0.29**	**(0.15; 0.42)**
Cognitive function	S-MMSE		−0.03	(−0.09; 0.03)	− 0.03	(− 0.08; 0.03)	0.05	(− 0.03; 0.12)	0.04	(− 0.03; 0.11)
Depression	CSDD		−0.02	(− 0.11; 0.07)	−0.02	(− 0.10; 0.06)	−0.09	(− 0.19; 0.01)	−0.08	(− 0.17; 0.01)
Functional status	KATZ ADL		**−0.34**	**(− 0.55; − 0.13)**	**−0.10**	**(− 0.16; − 0.04)**	−0.14	(− 0.40; 0.11)	−0.04	(− 0.12; 0.03)
Education	Length of formal education		0.02	(−0.06; 0.11)	0.01	(−0.04; 0.07)	0.02	(− 0.08; 0.12)	0.01	(− 0.05; 0.08)
Domestic environment	Home care (reference: ILTC)		**3.65**	**(2.88; 4.47)**	**0.58**	**(0.46; 0.70)**				
Informal carers										
Caregiver burden	ZBI						**0.10**	**(0.06; 0.14)**	**0.26**	**(0.15; 0.36)**
	NPI-Q-D						**−0.14**	**(−0.23; −0.04)**	**−0.19**	**(− 0.33; − 0.05)**
	CRA/Lack of family support						−0.04	(−0.13; 0.06)	−0.03	(− 0.10; 0.04)
Quality of life	GHQ-12						0.02	(−0.08; 0.12)	0.02	(−0.07; 0.11)
	EQ-5D-3 L						0.30	(−1.75; 2.34)	0.01	(−0.07; 0.10)
	EQ-5D VAS						−0.01	(− 0.04; 0.02)	−0.03	(− 0.11; 0.06)
	CRA/Impact on health						−0.09	(−0.25; 0.07)	−0.06	(− 0.16; 0.04)
Relationship to person with dementia	Child	(reference: husband)					−0.11	(−1.50; 1.28)	− 0.02	(− 0.24; 0.20)
	Wife						−0.30	(−1.91; 1.30)	− 0.05	(− 0.30; 0.21)
	Friend						−2.61	(−6.18; 0.97)	−0.41	(−0.98; 0.15)
	Other						−1.69	(−3.49; 0.07)	−0.27	(−0.54; 0.01)
Gender	Female (reference: male)						−0.15	(−1.31; 1.01)	− 0.02	(− 0.21; 0.16)

Statistical significant coefficients are printed in bold font

Value ranges of measures: see Additional file 1

#### Variables of people with dementia in model 1 and model 2

In Model 1, the care setting of the tested predictor variables showed the strongest influence on the difference between the self-rating and the proxy rating: B = 3.65; 95% CI (2.88; 4.42) corresponding with a standardised partial regression coefficient of β = 0.58, 95% CI (0.46; 0.70). This estimate indicates a clinically relevant difference between QoL-AD self- and proxy ratings in ILTC and HC, as previously defined (at a difference of more than three points in the total score of the QoL-AD*,* or more than half a standard deviation as expressed in z-scores).

BPSD of the person with dementia measured by the NPI-Q had the second strongest influence on the difference between the self-rating and the proxy rating in Model 1: B = 0.21, 95% CI (0.13; 0.29); β = 0.20, 95% CI (0.12; 0.28), and the strongest influence in Model 2: B = 0.30, 95% CI (0.15; 0.44); β = 0.29, 95% CI (0.15; 0.42). For both models, it was found that the higher the NPI-Q (more BPSD) the lower a proxy rated the QoL-AD*,* compared to a person with dementia.

The functional status measured by the KATZ ADL was negatively associated with the difference between the self- and the proxy ratings in both models. In Model 1 the influence of the functional status revealed statistical significance: B = − 0.34, 95% CI (− 0.55; − 0.13); β = − 0.10, 95% CI (− 0.16; − 0.04). In Model 2 the effect was smaller and did not reach statistical significance: B = − 0.14, 95% CI (− 0.40; 0.11); β = − 0.04, 95% CI (− 0.03; 0.11). Thus, the higher the KATZ ADL score (i.e., the less dependent the person with dementia) the higher the proxy rated the QoL-AD compared to the person with dementia.

No other tested variables of those with dementia in the two models revealed statistical significance.

#### Variables of informal carers in model 2

Two variables representing various aspects of caregiver burden revealed statistical significance, with opposite signs of the effect on the difference between self- and proxy ratings: for the ZBI*,* which represents general caregiver burden, B = 0.10, 95% CI (0.06; 0.14); β = 0.26, 95% CI (0.15; 0.36); and for the NPI-Q-D, which represents caregiver distress due to BPSD of the person with dementia, B = − 0.14, 95% CI (− 0.23; − 0.04); β = − 0.19, 95% CI (− 0.33; − 0.05). Thus, a higher ZBI (higher general burden) was associated with a lower proxy rating compared to the self-rating. A higher NPI-Q-D (higher caregiver distress related to BPSD) was associated with a higher proxy rating compared to the self-rating with the QoL-AD.

## Discussion

The aim of our study was to analyse the inter-rater agreement of the measures QoL-AD-SR and QoL-AD-PR, in terms of both the total score and the items, including a setting-specific analysis, and to identify factors associated with this agreement.

Our analysis suggests that proxies, i.e., informal carers and best informed professional carers, rate the QoL of those with dementia on average lower than those with dementia themselves. This is confirmed by previous studies that compare the performance of the two QoL-AD measures at the group level [16–43].

We found no acceptable inter-rater agreement (predefined range: +/− 3 scale points) of the QoL-AD measures in the whole sample. Comparing both settings and the means of differences, professional carers in ILTC rated the QoL on average one point less than the people with dementia, the informal carers in HC rated on average 4.7 points less. Nonetheless, no acceptable inter-rater agreement could be demonstrated in either HC or in ILTC when considering the Bland-Altman plots.

In almost all former studies [19, 20, 22, 26, 29, 31, 33, 36, 37, 39–41] the level of agreement at the QoL-AD total score was estimated using correlation analyses, which are not appropriate for agreement assessment. Bland-Altman plots were used in only two studies [25, 28]. Bosboom et al. [25] concluded that the agreement between self-rating and rating from the proxy-proxy perspective and from the person-proxy perspective is acceptable. However, this conclusion stems from a clear misunderstanding of the Bland-Altman plots method, and in particular LoA. The authors argued that only 2.5% (self-rating vs. proxy-proxy rating) and 5% (self-rating vs. person-proxy rating) of their Bland-Altman plots were beyond the LoA. However, LoA are derived from the distribution of the differences between paired observations, and should always include approximately 95% of these observations. This holds true for all Bland-Altman plots and LoA, independent of the actual agreement. For a valid conclusion, a predefined acceptable range for the differences would have been necessary. The actual LoA were − 8 to 6 points (self-rating vs. proxy-proxy rating) and − 9.5 to 15 points (self-rating vs. person-proxy rating). These LoA are far beyond an acceptable range. Zhao et al. [28] considered inter-rater agreement for the total score as fair (ICC = 0.58). They did not discuss their Bland-Altman plot, which even missed LoA. Using the given data from Zhao et al. [28], LoA ranges from − 7.3 to 13.9, indicating an unacceptable range of agreement.

Therefore, the QoL-AD proxy rating is not directly interchangeable with and cannot replace the QoL-AD self-rating of a person with dementia.

At the item level, we did not find any agreement better than poor except for the item ‘Marriage’, which achieved a fair agreement. The agreement on item level is comparable to the results of the studies by Conde-Sala et al. [33], Hoe et al. [40] and León-Salas et al. [31], which demonstrate mostly poor agreement on the item level. Chan et al. [29] and Wolak et al. [37] had a more positive conclusion, stating that agreement on the item level was largely good. Both studies inappropriately used correlation analysis for agreement rating.

We initially identified several factors that were hypothesised to be associated with the level of agreement and fitted two models. However, no factor importantly influenced the difference between self- and proxy ratings, except the setting (HC vs. ILTC).

The most frequently mentioned associated factor of those with dementia was BPSD [18, 20, 25, 28, 36]. In both fitted models we were able to confirm the strongest influence of all continuous variables for BPSD measured with the NPI-Q. Our results are in line with studies [18, 20, 25, 28, 36] that showed similar effect sizes and direction of effect.

The influence of the functional status or independence in activities of daily living is demonstrated in our analyses of Model 1. The level of agreement changed in such a way that the higher the dependence of the person with dementia (lower KATZ ADL score) the lower the QoL-AD rating of the proxy relative to that of the person with dementia. In Model 2, including only people with dementia living in HC, the effect was in the same direction but had a low magnitude and did not reach statistical significance. This confirms the results of Zhao et al. [28] and Zucchella et al. [18]. A possible explanation for the varying ratings could be the “disability paradox” introduced by Albrecht and Devlieger [66]. It means that many people with severe disabilities report a good QoL, although for external observers these people do not seem to be in good health. Another phenomenon in this context is the concept of response shift, defined as changes in the meaning of one’s self-evaluation of a target construct resulting from changes in internal standards, values, or conceptualisation [67]. Thus, to underestimate a person’s QoL compared to his or her own rating might also be a result of response shift.

Huang et al. showed that living in HC led to a lower difference of self- and proxy ratings compared to ILTC [17] (i.e., the proxy rating in HC was not as low as the self-rating in HC compared to ILTC ratings). This is the opposite of our results, but the ILTC proxy raters in the study by Huang et al. were also relatives, i.e., informal carers. In our analysis, we are not able to distinguish setting and proxies (staff ratings in ILTC and informal carer ratings in HC). Therefore, we cannot draw any conclusions as to whether the discrepancy results from differences according to the setting or from the proxy raters.

Unlike Bosboom et al. [25], Conde-Sala et al. [16, 19], Huang et al. [17] and Zhao et al. [28], we did not find any influence of the severity of cognitive impairment or depressive symptoms [18–20]. The level of education of the person with dementia, as stated by Huang et al. [17] and Tay et al. [20], could not explain the differences between measurements.

Caregiver burden has most often been identified in previous studies as an influencing factor of carers, and our analyses showed similar effects. Both measurements for caregiver burden (ZBI and NPI-Q-D) showed positive gradients on the difference in single regression models and highly correlated with each other (results not shown). The estimator for NPI-Q-D changed signs when combining both measurements in our predefined Model 2, because of multicollinearity. The extent of the standardised effect sizes of both estimators did not reach a relevant level, and are similar to previous results [18, 22, 28, 36]. The results of the studies by Huang et al. [36] and Schulz et al. [22] on the influence of the QoL of carers could not be confirmed in our analysis. No influence of the informal carer’s relationship to the person with dementia, as stated by Huang et al. [17], was shown. Finally, our analysis found no differences in terms of gender of the informal carer, unlike the findings of Conde-Sala et al. [19].

Overall, our fitted regression models covered only 8 and 13% of the observed variance of the difference between self- and proxy ratings. Therefore, it can be assumed that there are further unknown factors influencing the difference between self- and proxy ratings, which were not addressed by the data or our analysis.

### Strengths and limitations

The RTPC data for secondary data analysis provided us with access to a large European sample comprising QoL-AD self-ratings and proxy ratings.

However, setting-specific results should be interpreted with caution since ILTC settings might differ across countries. To ensure a widely representative sample 256 ILTC locations were included in the RTPC study; for the secondary analysis 476 professional carers rated the QoL.

Cross-cultural comparisons could not be conducted since the national sample sizes varied. However, we tested the assessment of the agreement between self- and proxy ratings of the QoL-AD total score on the German subsample (ILTC: *n* = 64; HC: *n* = 67). Again, no clear pattern of difference of the paired observations could be identified in the Bland-Altman plot.

## Conclusion

Our analysis showed that professional and informal carers appear to generate lower proxy ratings of QoL than those with dementia themselves. The assessment of the inter-rater agreement of the two measurement methods QoL-AD-SR and QoL-AD-PR revealed pronounced differences. Nevertheless, the QoL-AD benefits from good psychometric properties and the applicability to people with a wide range of dementia severity to rate themselves.

Our data indicate that the QoL-AD self- and proxy ratings are not directly interchangeable due to the inter-rater gap. Thus, QoL-AD-PR provides a complementary perspective rather than a substitute for self-rating. In particular, a mix of self-rating and proxy rating may be biased. From a clinical point of view, our study suggests that either only one rating method should be performed or both rating methods parallel with separate analyses grouped in self- and proxy ratings. Self-ratings should be applied whenever possible. It is also required to report transparently who has responded to the QoL-AD.

An implication for future research would be to compare the QoL-AD-SR with the corresponding ratings of several proxy groups such as informal carers and professional carers. This would allow comparison of the levels of inter-rater agreement between the person with dementia and various proxies, and the agreement among the latter. Hereby, the impact of the setting on the level of agreement might be taken into account more specifically.

## Additional files

Additional file 1: Variables and measures from the RightTimePlaceCare study data set, based on factors associated with the level of agreement between self- and proxy ratings identified in the empirical literature. (PDF 136 kb)

Additional file 2: Flow chart of study sample selection. (PDF 104 kb)

## Abbreviations

BPSD: Behavioural and psychological symptoms of dementia
CI: Confidence interval
CRA: Caregiver Reaction Assessment
CSDD: Cornell Scale for Depression in Dementia
EQ-5D VAS: European Quality of Life-Visual Analog Scale
EQ-5D-3 L: European Quality of Life Scale
GHQ-12: General Health Questionnaire
HC: Formal home care
ICC: Intraclass correlation coefficient
ILTC: Institutional long-term nursing care
KATZ ADL: Katz Index of Independence in Activities of Daily Living
LoA: Limits of agreement
NPI-Q: Neuropsychiatric Inventory Questionnaire
NPI-Q-D: Neuropsychiatric Inventory Questionnaire-Caregiver Distress
PR: Proxy rating
QoL: Quality of life
QoL-AD: Quality of Life in Alzheimer’s Disease scale
QoL-AD-PR: Quality of Life in Alzheimer’s Disease Proxy Rating scale
QoL-AD-SR: Quality of Life in Alzheimer’s Disease Self-Rating scale
RTPC: The European RightTimePlaceCare study
SD: Standard deviation
S-MMSE: Standardized Mini-Mental State Examination
SR: Self-rating
ZBI: Zarit Burden Index
