# Supplementary material for: Inter-rater agreement of the Quality of Life-Alzheimer’s Disease (QoL-AD) self-rating and proxy rating scale: secondary analysis of RightTimePlaceCare data
Source: Health Qual Life Outcomes. 2018 Jun 28;16:131. doi: 10.1186/s12955-018-0959-y (PMC6022444; doi:10.1186/s12955-018-0959-y)
Supplement: Supplementary file 1 — Variables and measures from the RightTimePlaceCare study data set, based on factors associated with the level of agreement between self- and proxy ratings identified in the empirical literature. (PDF 136 kb) [file 12955_2018_959_MOESM1_ESM.pdf]

Additional file 1: Variables and measures from the RightTimePlaceCare study data set, based on factors associated with the level of agreement between self- and proxy ratings identified in the empirical literature

Findings of the empirical literature				Measurements of the RightTimePlaceCare study					
Factors associated with the level of agreement			Studies	Outcome	Measure/ variable	Subscales/ items	Setting: rater	Value range	Interpretation
People with dementia	1.	Behavioural and psychological symptoms of dementia (BPSD)	n = 5 [18, 20, 25, 28, 36]	Challenging behaviour	Neuropsychiatric Inventory Questionnaire (**NPI-Q**) [49, 50]	12 items in 12 behavioural domains: delusions, hallucinations, agitation/aggression, depression/dysphoria, anxiety, euphoria, apathy, disinhibition, irritability, aberrant motor behaviour, sleep and night-time behaviour change, appetite and eating change	ILTC: professional carers<br>HC: informal carers	0 – 36	Higher scores indicate the presence of more (severe) neuropsychiatric symptoms
		Cognitive impairment or severity of dementia	n = 5 [16, 17, 19, 25, 28]	Cognitive function	Standardized Mini Mental-State Examination **(S-MMSE)** [47, 51]	30-item assessment of global cognitive function with domains such as orientation, concentration, attention, memory, recall and calculation	ILTC+HC: person with dementia or routine data	0 – 30	Lower scores indicate severe cognitive impairment<br><br>Sometimes used as a surrogate method for the staging of dementia: severe=0–10, moderate=11–20, mild=21–25, questionable=26–29, no=30 [52]<br>→ However, cut-off points to assist in the diagnosis of dementia or to determine dementia severity should be interpreted cautiously [1].
	2.	Depression	n = 3 [18–20]	Depressive symptoms	Cornell Scale for Depression in Dementia (**CSDD**) [53]	19 items covering 5 dimensions: mood-related signs, behavioural disturbance, physical signs, cyclic functions, ideational disturbance	ILTC: professional carers<br>HC: informal carers	0 – 38	Higher scores indicate the presence of increased depressive symptoms<br><br>> 6 points: presence of considerable depressive symptoms

Findings of the empirical literature				Measurements of the RightTimePlaceCare study					
		**Factors associated with the level of agreement**	**Studies**	**Outcome**	**Measure/ variable**	**Subscales/ items**	**Setting: rater**	**Value range**	**Interpretation**
	3.	Functional status: independence	n = 2 [18, 28]	Functional status	Katz Index of Independence in Activities of Daily Living **(KATZ ADL)** [54]	6 activity domains:<br>bathing, dressing, toileting, transferring, continence, feeding	ILTC: professional carers<br>HC: informal carers	0 – 6	Higher scores indicate more independency in performing activities of daily living<br>6 points: independent, 3–5 points: moderate dependency, 0–2 points: severe dependency
		Anosognosia	n = 2 [19, 21]	Not measured					
		Quality of person with dementia-carer relationship	n = 2 [17, 36]	Not measured					
		Educational background	n = 2 [17, 20]	Sociodemographic data: length of formal education (years)			ILTC+HC: informal carers		
	4.	Domestic environment/ person with dementia living at home	n = 1 [17]	Sociodemographic data: care setting (HC vs. ILTC)					
**Carers**	1.	Caregiver burden	n = 4 [18, 22, 28, 36]	General caregiver burden	Zarit Burden Inventory (**ZBI**) [55]	22 items covering 5 domains of general caregiver burden:<br>burden in relationship, emotional wellbeing, social and family life, finances, loss of control over one's life	ILTC+HC: informal carers	0 – 88	Higher scores indicate higher perceived general caregiver burden
				Caregiver distress due to BPSD of the person with dementia	Neuropsychiatric Inventory Questionnaire-Caregiver Distress (**NPI-Q-D**) [49, 50]	12 items in 12 behavioural domains of caregiver distress associated with symptoms of BPSD of the person with dementia: delusions, hallucinations, agitation/aggression, depression/dysphoria, anxiety, euphoria, apathy, disinhibition, irritability, aberrant motor behaviour, sleep and night-time behaviour change, appetite and eating change	HC only: informal carers	0 – 60	Higher scores indicate higher caregiver distress related to BPSD of the person with dementia

Findings of the empirical literature				Measurements of the RightTimePlaceCare study						
**Factors associated with the level of agreement**			**Studies**	**Outcome**	**Measure/ variable**	**Subscales/ items**	**Setting: rater**	**Value range**	**Interpretation**
				Reactions of informal carers to the care of chronically ill persons	Caregiver Reaction Assessment (**CRA**) [56]	Subscale: **CRA-Lack of family support**	ILTC+HC: informal carers	5 – 25	Higher scores indicate a more negative influence on carers' experience
	2.	Depression of the carer	n = 3 [17, 22]	Not measured					
	3.	Quality of life of the carer	n = 2 [22, 36]	Health-related quality of life	European Quality of Life Scale **(EQ-5D)** [57, 58]	**EQ-5D-3L** 5 items about 5 health dimensions: mobility, self-care, usual activities, pain/discomfort, anxiety/depression	ILTC+HC: informal carers	-0,594 – 1	Higher scores indicate better health-related quality of life
						**EQ-5D VAS** Visual Analogue Scale (VAS) to record self-rated health status	ILTC+HC: informal carers	0 – 100	Higher scores indicate a higher self-rated health status of the informal carer
				Psychological well-being	General Health Questionnaire 12-item version (**GHQ-12**) [59]	12 items	HC: informal carers	0 – 36	Higher scores indicate a less psychological well-being
				Reactions of informal carers to the care of chronically ill persons	Caregiver Reaction Assessment (**CRA**) [56]	Subscale: **CRA-Impact on health**	ILTC+HC: informal carers	4 – 20	Higher scores indicate a more negative influence on carers' experience due to their own health problems
	4.	Professional carer being a Primary Nurse	n = 1 [27]	Not measured					
		Kinship status	n = 1 [17]	Sociodemographic data: relationship to person with dementia			ILTC+HC: informal carers		

Findings of the empirical literature					Measurements of the RightTimePlaceCare study					
**Factors associated with the level of agreement**				**Studies**	**Outcome**	**Measure/ variable**	**Subscales/ items**	**Setting: rater**	**Value range**	**Interpretation**
			Gender of the carer	n = 1 [19]	Sociodemographic data: gender			ILTC+HC: informal carers		
			Income of the carer	n = 1 [22]	Not measured					

ILTC: institutional long-term nursing care; *HC:* professional home care.

All references mentioned in the table are cited in the ‘References’ section of the main publication.
