# Supplementary figures and images for: Inter-rater agreement of the Quality of Life-Alzheimer’s Disease (QoL-AD) self-rating and proxy rating scale: secondary analysis of RightTimePlaceCare data
Source: Health Qual Life Outcomes. 2018 Jun 28;16:131. doi: 10.1186/s12955-018-0959-y (PMC6022444; doi:10.1186/s12955-018-0959-y)

# Additional file 2: Flow chart of study sample selection

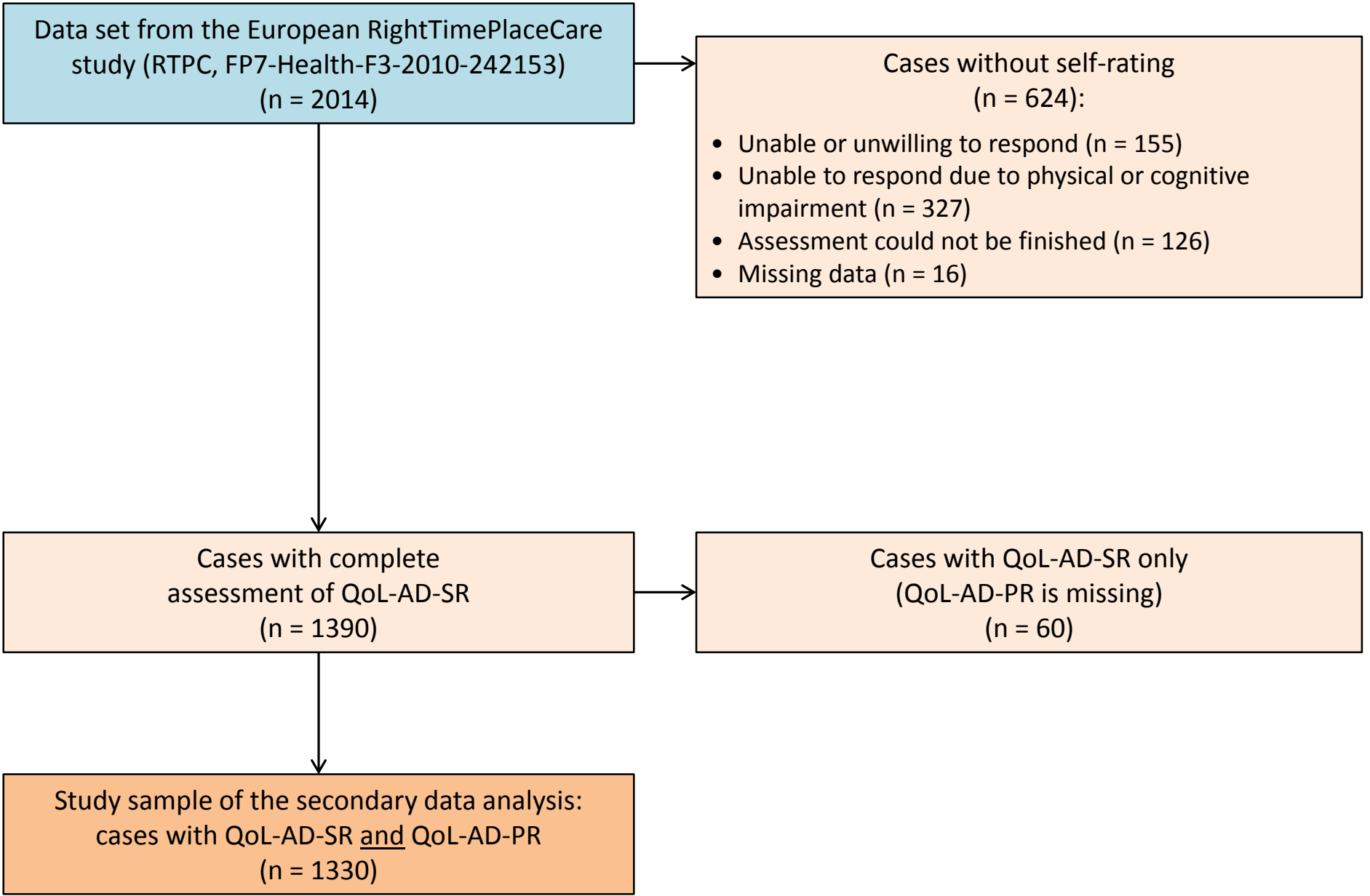

Supplement: Supplementary file 2 — Flow chart of study sample selection. (PDF 104 kb) [file 12955_2018_959_MOESM2_ESM.pdf]
